# Supplementary material for: COZOID: contact zone identifier for visual analysis of protein-protein interactions
Source: BMC Bioinformatics. 2018 Apr 6;19:125. doi: 10.1186/s12859-018-2113-6 (PMC5889581; doi:10.1186/s12859-018-2113-6)
Supplement: Supplementary file 2 — Software build. Executable binary file of the software tool. (ZIP 118,784 kb) [file 12859_2018_2113_MOESM2_ESM.zip › cozoid/platform/docs/testng-6.5.1-javadoc.zip!/org/testng/IResultMap.html]

IResultMap (TestNG)


---


|  |  |  |
| --- | --- | --- |
| **Overview** \| **Package** \| **Class** \| **Tree** \| **Deprecated** \| **Index** \| **Help** | |  |
| **PREV CLASS**   **NEXT CLASS** | **FRAMES**    **NO FRAMES**     **All Classes** |  |
| SUMMARY: NESTED \| FIELD \| CONSTR \| METHOD | DETAIL: FIELD \| CONSTR \| METHOD |  |


---


## org.testng Interface IResultMap

**All Superinterfaces:**: java.io.Serializable

---

``` public interface IResultMap extends java.io.Serializable ```

---

| **Method Summary** | |
| --- | --- |
| `void` | `addResult(ITestResult result, ITestNGMethod method)` |
| `java.util.Collection<ITestNGMethod>` | `getAllMethods()` |
| `java.util.Set<ITestResult>` | `getAllResults()` |
| `java.util.Set<ITestResult>` | `getResults(ITestNGMethod method)` |
| `void` | `removeResult(ITestNGMethod m)` |
| `int` | `size()` |

**Method Detail**

### addResult

```
void addResult(ITestResult result,
               ITestNGMethod method)
```

---


### getResults

```
java.util.Set<ITestResult> getResults(ITestNGMethod method)
```

---


### getAllResults

```
java.util.Set<ITestResult> getAllResults()
```

---


### removeResult

```
void removeResult(ITestNGMethod m)
```

---


### getAllMethods

```
java.util.Collection<ITestNGMethod> getAllMethods()
```

---


### size

```
int size()
```


---


|  |  |  |
| --- | --- | --- |
| **Overview** \| **Package** \| **Class** \| **Tree** \| **Deprecated** \| **Index** \| **Help** | |  |
| **PREV CLASS**   **NEXT CLASS** | **FRAMES**    **NO FRAMES**     **All Classes** |  |
| SUMMARY: NESTED \| FIELD \| CONSTR \| METHOD | DETAIL: FIELD \| CONSTR \| METHOD |  |


---
